# Precision meets generalization: Enhancing brain tumor classification via pretrained DenseNet with global average pooling and hyperparameter tuning

**DOI:** 10.1371/journal.pone.0307825

**Published:** 2024-09-06

**Authors:** Najam Aziz, Nasru Minallah, Jaroslav Frnda, Madiha Sher, Muhammad Zeeshan, Amara Haroon Durrani

**Affiliations:** 1 Department of Computer Systems Engineering, University of Engineering and Technology(UET), Peshawar, Khyber Pakhtunkhwa, Pakistan; 2 National Center for Big Data and Cloud Computing (NCBC), University of Engineering and Technology, Peshawar, Khyber Pakhtunkhwa, Pakistan; 3 Department of Quantitative Methods and Economic Informatics, Faculty of Operation and Economics of Transport and Communication, University of Zilina, Zilina, Slovakia; 4 Department of Telecommunications, Faculty of Electrical Engineering and Computer Science, VSB - Technical University, Ostrava-Poruba, Czechia; 5 Department of Radiology, Hayatabad Medical Complex, Peshawar, Pakistan; Najran University College of Computer Science and Information Systems, SAUDI ARABIA

## Abstract

Brain tumors pose significant global health concerns due to their high mortality rates and limited treatment options. These tumors, arising from abnormal cell growth within the brain, exhibits various sizes and shapes, making their manual detection from magnetic resonance imaging (MRI) scans a subjective and challenging task for healthcare professionals, hence necessitating automated solutions. This study investigates the potential of deep learning, specifically the DenseNet architecture, to automate brain tumor classification, aiming to enhance accuracy and generalizability for clinical applications. We utilized the Figshare brain tumor dataset, comprising 3,064 T1-weighted contrast-enhanced MRI images from 233 patients with three prevalent tumor types: meningioma, glioma, and pituitary tumor. Four pre-trained deep learning models—ResNet, EfficientNet, MobileNet, and DenseNet—were evaluated using transfer learning from ImageNet. DenseNet achieved the highest test set accuracy of 96%, outperforming ResNet (91%), EfficientNet (91%), and MobileNet (93%). Therefore, we focused on improving the performance of the DenseNet, while considering it as base model. To enhance the generalizability of the base DenseNet model, we implemented a fine-tuning approach with regularization techniques, including data augmentation, dropout, batch normalization, and global average pooling, coupled with hyperparameter optimization. This enhanced DenseNet model achieved an accuracy of 97.1%. Our findings demonstrate the effectiveness of DenseNet with transfer learning and fine-tuning for brain tumor classification, highlighting its potential to improve diagnostic accuracy and reliability in clinical settings.

## Introduction

Brain tumors pose a significant health challenge worldwide due to their high fatality rates and few available treatments options. They can be divided into two types: primary tumors and secondary tumors, and they originate from abnormal growth in brain cells [1]. About 70% of brain tumor cases are primary tumors; the remaining 30% are secondary tumors, which start in other parts of the body and then spread to the brain. The most common forms of primary brain tumors are pituitary, meningioma, and glioma [2, 3].

Accurate and early detection of these tumors is crucial for improving patient outcomes. However, manual detection and diagnosis from magnetic resonance imaging (MRI) scans are challenging, as they rely heavily on radiologists’ expertise and are prone to subjectivity and errors due to the complex nature of brain tumor presentations [4, 5]. To address these challenges, Deep learning (DL)-based automated brain tumor classification systems have drawn a lot of interest as a solution to these problems. In the field of medical image analysis, DL algorithms—in particular, Convolutional Neural Networks (CNNs)—have shown impressive performance, especially for problems like brain tumor classification using Medical Resonance (MR) images [6]. However, existing literature [7–10] mainly emphasizes accuracy metrics without adequately addressing model generalization to new, unseen data. This gap is crucial, as the reliability of brain tumor classification models in clinical settings depends on their generalization capabilities. Furthermore, while various deep learning architectures have been explored, comprehensive comparisons and optimizations, particularly involving DenseNet, are limited. Our research addresses crucial gap in the existing literature by not only focusing on achieving high accuracy but also emphasizing the robustness and generalizability of the model in clinical settings.

This research builds upon the foundation let by Prakash et al [7], which is set as a benchmark study, and exhibits significant shortcomings in terms of model generalization, highlighting the need for more robust approaches. Our study leverages the DenseNet architecture and the same Figshare brain tumor dataset utilized by [7] to create a robust and accurate DL-based model for brain tumor classification [11, 12]. The dataset provides a comprehensive collection of T1-weighted contrast-enhanced MRI images, enabling the training of a DenseNet and other CNN based alogrithms [13] for accurate tumor classification. By leveraging data augmentation, regularization techniques and hyperparameter optimization, we aim to improve the accuracy and generalization of the densenet model for brain tumor diagnosis in clinical settings. The key contributions of this study to the existing literature are as follows;

- Comprehensive Model Comparison: We systematically compared the performance of DenseNet with other state-of-the-art pre-trained models, including ResNet, EfficientNet, and MobileNet, providing a thorough evaluation of their efficacy in brain tumor classification using the Figshare dataset.
- Fine-Tuning Approach: We implemented a unique fine-tuning methodology for the DenseNet model, incorporating advanced regularization techniques such as data augmentation, batch normalization and dropout with global average pooling to address overfitting and improve generalization on unseen data.
- Enhanced Generalization: By optimizing hyperparameters and employing rigorous training and validation protocols, we demonstrated significant improvements in the generalization capabilities of the DenseNet model, making it more robust and reliable for real-world clinical applications.

By integrating recent advancements in deep learning and transfer learning, our study aims to develop a reliable and efficient brain tumor classification system that can assist healthcare professionals in making informed diagnostic decisions, ultimately leading to improved patient care.

The proposed methodology for brain tumor classification in this study represents a significant advancement in deep learning techniques for medical imaging. Unlike previous works that often focused on a single model architecture, this study rigorously compares and optimizes four popular architectures, with DenseNet emerging as the most effective choice. What sets this approach apart is its emphasis on enhancing both performance and generalization capabilities. By incorporating regularization techniques tailored for DenseNet and carefully adjusting hyperparameters, the methodology addresses challenges such as overfitting while improving the model’s ability to generalize to unseen data. This comprehensive approach not only achieves high accuracy but also ensures robustness and reliability in real-world applications.

## Literature review

Brain tumor classification holds significant importance in medical image analysis, and recent advancements in deep learning have significantly impacted this field. These advancements empower the extraction of intricate features and patterns from large datasets [14]. By training deep learning models on extensive collections of medical images, encompassing modalities such as MRI, the potential arises to create exceptionally accurate and efficient algorithms for the classification of brain tumors. These algorithms possess the capability to enhance clinical decision-making, assist in accurate diagnosis, and contribute to improved patient outcomes.

Convolutional neural networks (CNNs) have been at the forefront of this research, demonstrating significant potential in distinguishing between brain tumor classes. For instance, Togacar et al. [15] introduced BrainMRNET, achieving an impressive accuracy of 96.05% in binary classification. Similarly, Mzoughi et al. [16] effectively differentiated between High-Grade Glioma (HGG) and Low-Grade Glioma (LGG) with a high accuracy of 96.49%. Moreover, [17, 18] in their studies successfully diagnosed brain tumors with a CNN, achieving a high accuracy of 95.49% and 96.00%. Study by Khawaldeh et al. [19] improved the AlexNet CNN model for multi-class classification, achieving a notable accuracy of 91% in classifying both normal/abnormal and high-grade/low-grade glioma tumors. These studies emphasize the relevance of CNNs in clinical applications for brain tumor diagnosis.

More complex CNN architectures, such as Capsule Networks [20] and Genetic Algorithms [21], have also shown promise in improving classification performance. Kurup et al. [22] showcased the effectiveness of CapsNet, achieving an impressive accuracy of 92.6%. These advancements show the significance of exploring novel approaches to improve the accuracy of brain tumor classification models.

In addition to CNNs, alternative models have been investigated for brain tumor classification as well. Studies have investigated the efficacy of K-Nearest Neighbors (KNN), Support Vector Machines (SVM), extreme learning machines (ELM), and hybrid approaches like SVM-KNN [11, 23–26]. These investigations have reported accuracies ranging from 84.19% to 95.56%, highlighting the potential of diverse methodologies in this domain.

Transfer learning has emerged as a successful approach in improving brain tumor classification performance. Researchers have leveraged pre-trained CNN models and knowledge from large-scale datasets to enhance accuracy [27]. For instance, Sajjad et al. [28] achieved a commendable accuracy of 94.5% by fine-tuning a VGG-19 model, while Waghmere et al. [29] obtained an accuracy of 95.71% using VGG16. These findings show the effectiveness of transfer learning in leveraging existing knowledge for improved classification accuracy.

These studies show that brain tumor classification research has demonstrated the potential of CNNs, transfer learning and deep learning architectures. Significant progress has been made in improving classification accuracy through innovative approaches, such as multiclass classification, transfer learning, and model selection.

However, despite these significant advancements in deep learning, overfitting remains a critical challenge in developing robust models for brain tumor classification. Recent studies by [7–10] report high accuracies but reveal substantial gaps between training and validation performance, suggesting overfitting. The models employed struggled to generalize to unseen data. This persistent issue underscores the necessity for more robust strategies to ensure reliable model performance in real-world clinical applications.

## Materials and methods

### Dataset description

**Fig 1:**
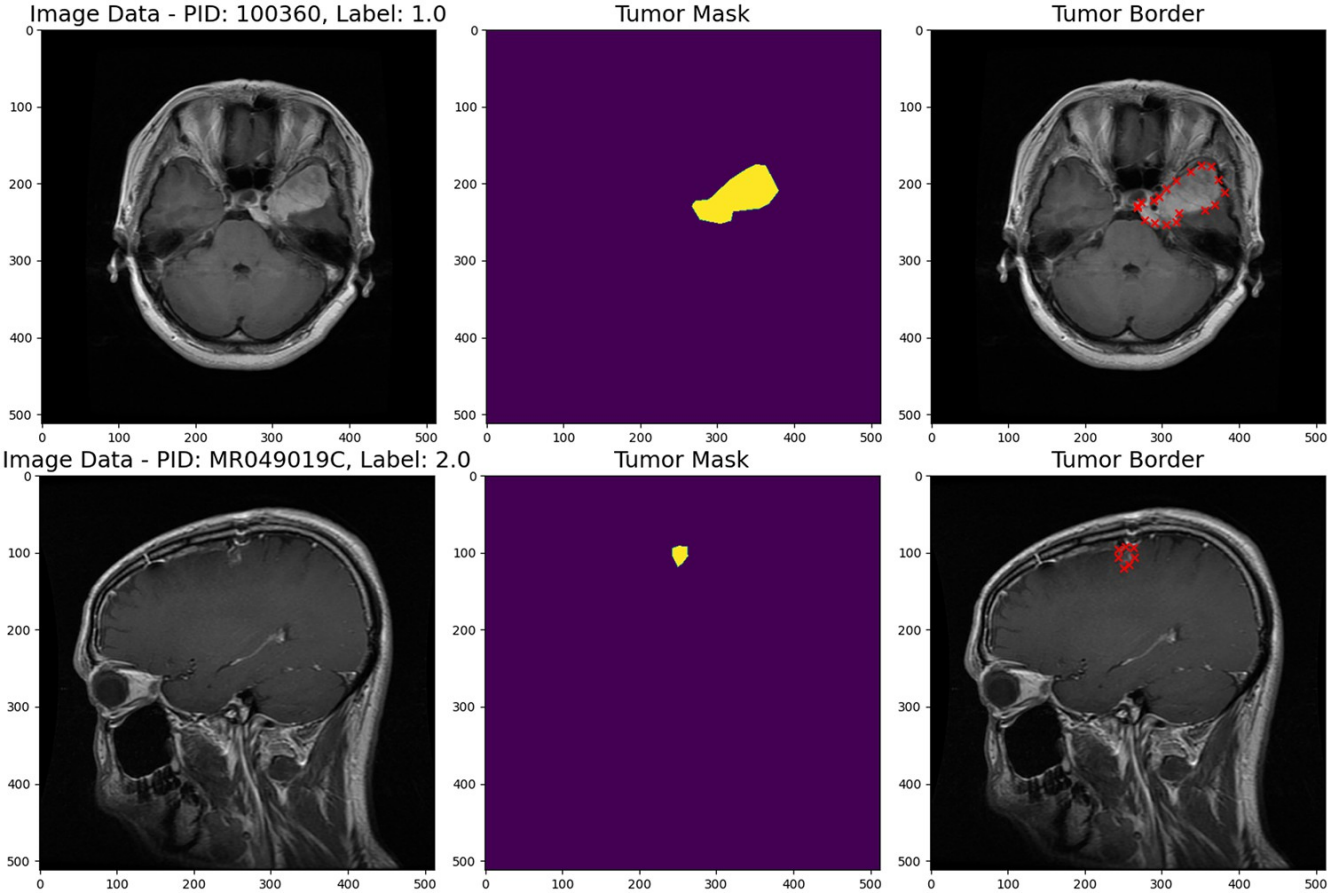
Sample data from the dataset.

The brain tumor dataset used for this study has 3,064 109 T1-weighted contrast-enhanced images from 233 patients. The dataset has three distinct types of brain tumors: meningioma (708 slices), glioma 111 (1,426 slices), and pituitary tumor (930 slices). Furthermore, the dataset consists of 112 files organized in MATLAB (.mat) structure. This dataset has been previously used in research studies, specifically in the papers by Cheng et al [11, 12]. The dataset is designed to support research and analysis in the field of medical image processing and brain tumor classification. Samples of the dataset can be seen in Fig 1. In this study, the Figshare brain tumor dataset is split into train and test sets with an 80:20 ratio. This means that 80% of the data is used for training the models, while the remaining 20% is reserved for testing.

### Convolutional neural network

The human visual system provides bases for Convolutional Neural Networks (CNNs), and allow them to automatically identify patterns within intricate images like MRI scans.

This eliminates the necessity for handcrafting features, a laborious step in conventional image analysis. CNNs achieve this through several key architectural components including convolutional layers, pooling layers, and activation functions, in addition to other important elements. In the context of brain tumor categorization, CNNs analyze MR scans, distinguishing tumor types and providing crucial diagnostic insights. By exploring diverse CNN models such as ResNet, EfficientNet, MobileNet and DenseNet, while leveraging transfer learning, this research contributes to the advancement of deep learning for accurate classification of brain tumors, potentially leading to improved healthcare outcomes.

### Transfer learning

Transfer learning has revolutionized medical image analysis by harnessing the knowledge acquired by pre-trained neural networks.

**Fig 2:**
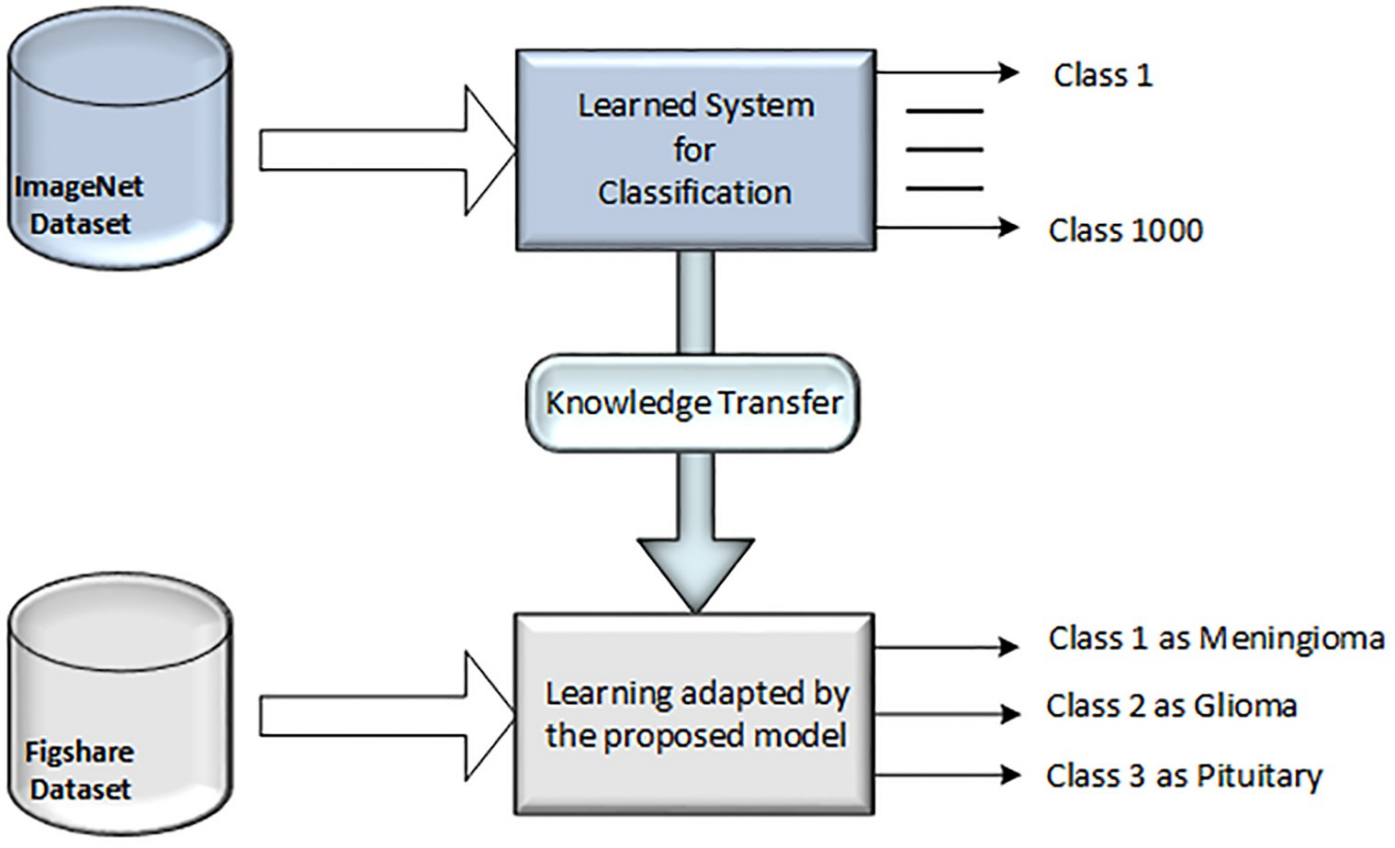
Knowledge transfer from ImageNet to Figshare.

In our experimental setup, we initialized the weights of these models using the pre-trained weights obtained after extensive training with large-scale image dataset, the ImageNet. Subsequently, we fine-tuned the classification layer of these models on the Figshare dataset, which contains annotated brain tumor images, to adapt the learned features to the specific classification task. By leveraging transfer learning, we aimed to capitalize on the representational power of these pre-trained models while tailoring them to the nuances of brain tumor classification. The Fig 2 shows the process of knowledge transfer from the ImageNet to Figshare data.

Initially, the four pretrained CNN architectures are implemented and the performance of these model over the brain tumor classification task are compared. Brief description of the utilized pretrained models and their performance are provided as follow.

### Employed models

#### ResNet

ResNet is a deep CNN-based model introduced by He et al. [30]. The use of residual blocks, which addresses the issue of the gradient vanishing in deep networks, is one of its distinguishing features. The presence of residual connections facilitates the training of deeper architectures by allowing the model to learn residual functions with regard to the layer inputs.

#### EfficientNet

EfficientNet is a family of CNN architectures proposed by Tan et al. [31], which attain cutting-edge performance by systematically scaling network complexity and resolution. The core idea behind EfficientNet is to use a compound scaling method that uniformly scales all dimensions of the network to achieve better performance.

#### MobileNet

With a focus on embedded vision and mobility, Howard et al. [32], developed MobileNet a lightweight CNN architecture. MobileNet utilizes specialized convolutions to lower computational overhead while still delivering competitive accuracy. This architecture is particularly suitable for resource-constrained environments due to its low memory footprint and computational efficiency.

#### DenseNet—The base model

**Fig 3:**
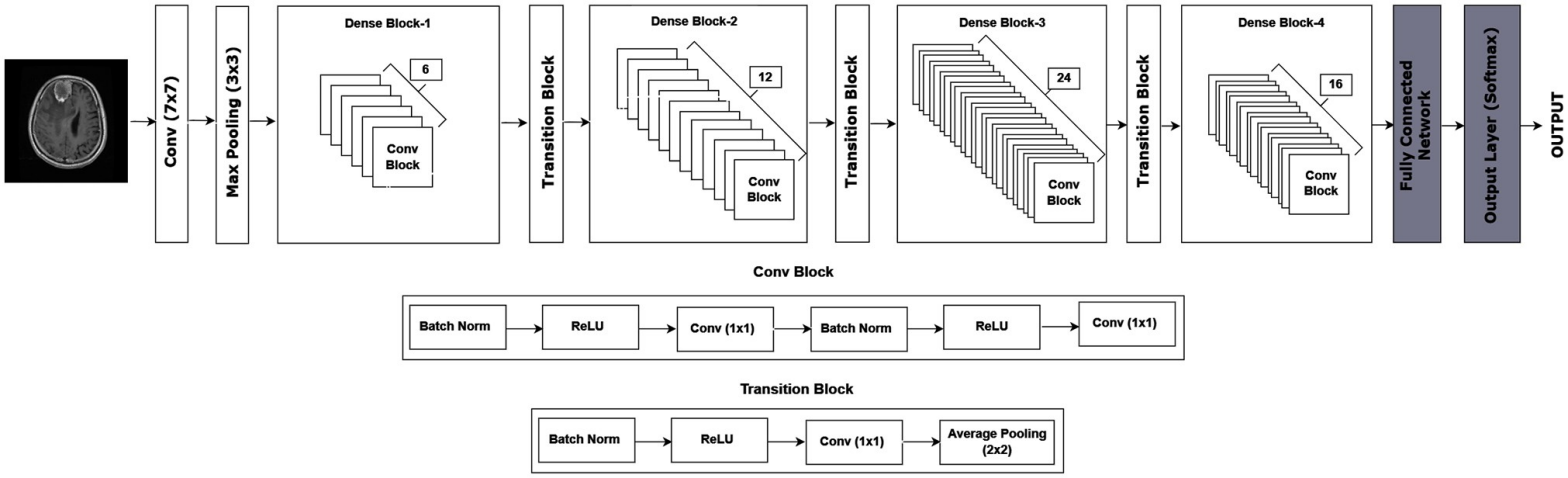
DenseNet base model.

DenseNet first proposed by Huang et al. [33] is a deep CNN-based model that has shown incredible performance in various vision applications. The architecture consists of several dense blocks, wherein each block encompasses multiple convolutional layers with direct connections between them. extraction. In our experimental setup, the DenseNet model demonstrated great performance in comparison to other three. Hence, choosen as base model for further experimentation and improvements. The DenseNet base model architecture is given in Fig 3.

### Enhanced DenseNet

**Fig 4:**
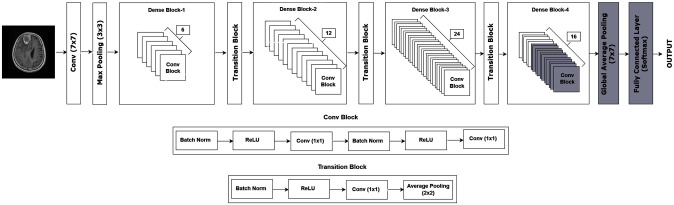
DenseNet customized model.

To enhance the effectiveness of the base DenseNet model, a customized architecture was implemented which is illustrated in Fig 4. For brain tumor classification, transfer learning was employed by freezing the initial layers, while unfreezing the last 11 layers of DenseNet architecture. This approach allows the model to build upon its existing knowledge while learning new, specific features for brain tumors. In brain tumor classification, the exact position of the tumor within the MRI scan is less important than its overall presence and characteristics. Hence, we incorporated a global average pooling(GAP) layer at the end of the network. By incorporating GAP, we ensured the model captured these crucial features for accurate classification.

#### Training process and hyperparameters

To achieve optimal performance while maintaining computational efficiency, a comprehensive training strategy was employed. A batch size of 128 was chosen, achieving a balance between training speed and the model’s ability to learn from the data. To prevent overfitting, a learning rate of 0.0001 was used, allowing for precise adjustments to the model’s weights. Additionally, training for 100 epochs ensured that the model learned sufficiently without excessive iterations, as determined through validation monitoring.

Data augmentation techniques, such as random flips, rotations, and zooms, were employed to increase the diversity of the training data. This process helps the model generalize better to unseen examples. Dropout, with a rate of 50%, and batch normalization were further incorporated to mitigate overfitting and stabilize the training process.

The Adam optimizer, known for its efficiency and effectiveness in deep learning, was chosen with the aforementioned learning rate. This combination facilitated the optimization process and ensured model convergence.

This approach capitalizes on transfer learning from the well-established DenseNet architecture. By fine-tuning specific layers, the model adapted its knowledge to the brain tumor classification task. The rigorous hyperparameter tuning and regularization techniques empowered the model to achieve a balance between efficiency and performance.

#### Model selection criteria

The generalizability of the trained models is assessed using a separate validation dataset not used during training. Performance metrics such as accuracy, precision, recall, and F1-score were evaluated to measure the model’s ability to classify brain tumors on unseen data. By carefully selecting hyperparameters, employing regularization techniques, and evaluating performance on unseen data, this study ensures the generalizability of the trained models for real-world applications in brain tumor detection. The comprehensive evaluation on a validation dataset demonstrates the model’s robustness and reliability, making it suitable for clinical applications where accurate and consistent performance is crucial.

## Experimental results and discussions

The experimentation results of all the employed models in this study are provided and discussed below.

### ResNet

**Fig 5:**
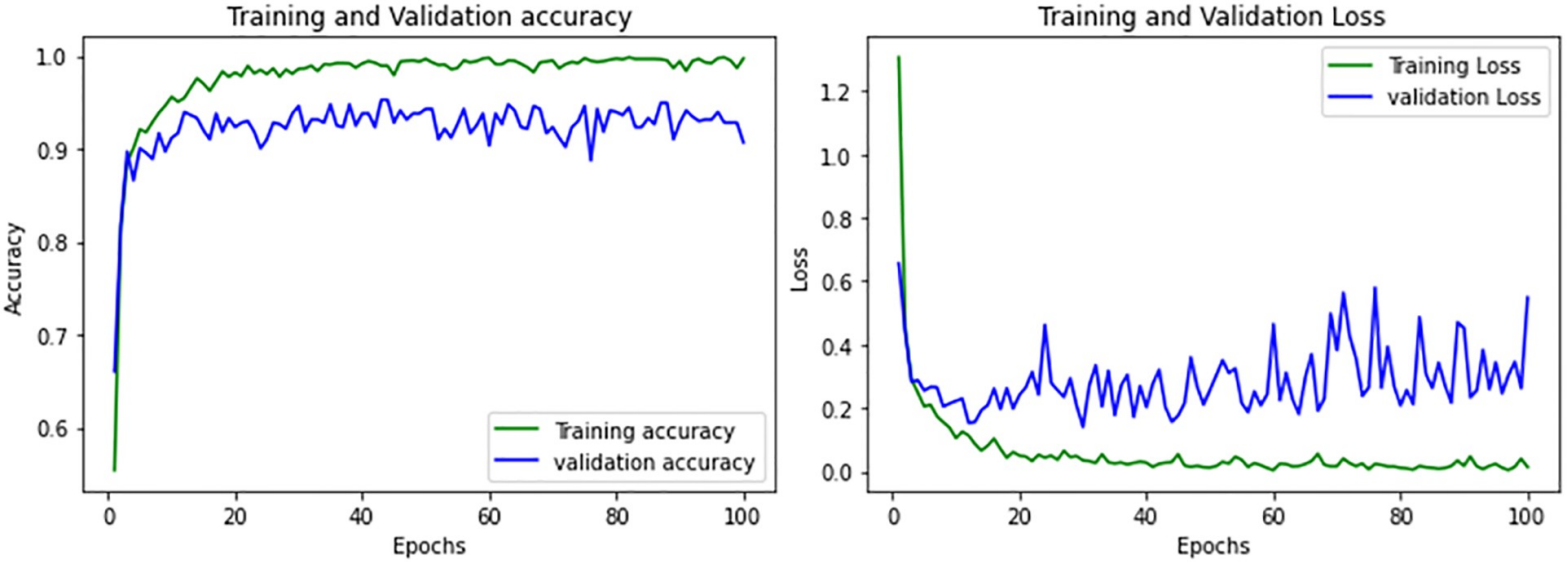
Performance curves of ResNet.

The accuracy and validation curves in Fig 5 and evaluation metrics demonstrated in Table 1 shows the effectiveness of the ResNet architecture. ResNet exhibits good training accuracy, signaling effective learning. However, validation accuracy suggests potential overfitting. While achieving acceptable overall classification accuracy (0.91), ResNet’s performance varies across tumor classes, with room for optimization, particularly in addressing overfitting concerns.

**Table 1:** Classification report of ResNet.

Tumor Class	Precision	Recall	F1-score	support
Meningioma	0.87	0.82	0.85	142
Glioma	0.98	0.9	0.94	306
Pituitary	0.82	0.99	0.9	165
Accuracy			0.91	613
Macro Average	0.89	0.91	0.9	613
Weighted Average	0.91	0.91	0.91	613

### EfficientNet

**Fig 6:**
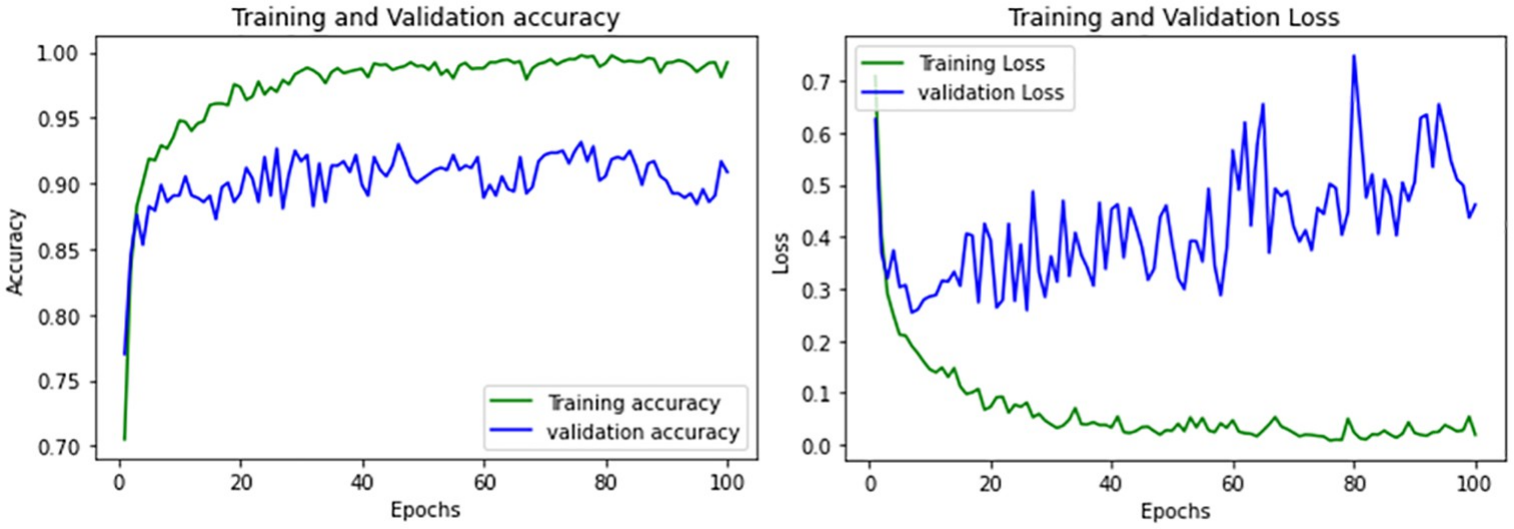
Performance curves of EfficientNet.

The performance graphs provided in Fig 6 and classification reports in Table 2 shows that EfficientNet has more severe overfitting issue and degraded performance over the validation set.

**Table 2:** Classification report of EfficientNet.

Tumor Class	Precision	Recall	F1-score	support
Meningioma	0.89	0.77	0.83	142
Glioma	0.95	0.93	0.94	306
Pituitary	0.85	0.99	0.92	165
Accuracy			0.91	613
Macro Average	0.9	0.9	0.89	613
Weighted Average	0.91	0.91	0.91	613

### MobileNet

**Fig 7:**
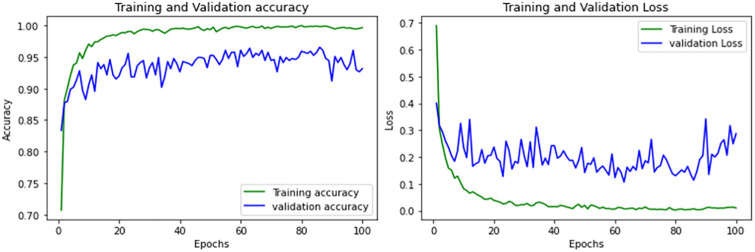
Performance curves of MobileNet.

MobileNet, a lightweight CNN architecture, achieves an accuracy of 0.93 in brain tumor classification. It exhibits potential overfitting, similar to ResNet and EfficientNet with a gap between training and validation accuracies. While showing strengths in certain classes, it requires optimization for others. Improvements targeting overfitting and class-specific performance are suggested for enhancement. The performance of MobileNet is shown in Fig 7 and Table 3.

**Table 3:** Classification report of MobileNet.

Tumor Class	Precision	Recall	F1-score	support
Meningioma	0.94	0.82	0.88	142
Glioma	0.97	0.95	0.96	306
Pituitary	0.87	1	0.93	165
Accuracy			0.93	613
Macro Average	0.93	0.92	0.92	613
Weighted Average	0.93	0.93	0.93	613

### DenseNet—Base model

**Fig 8:**
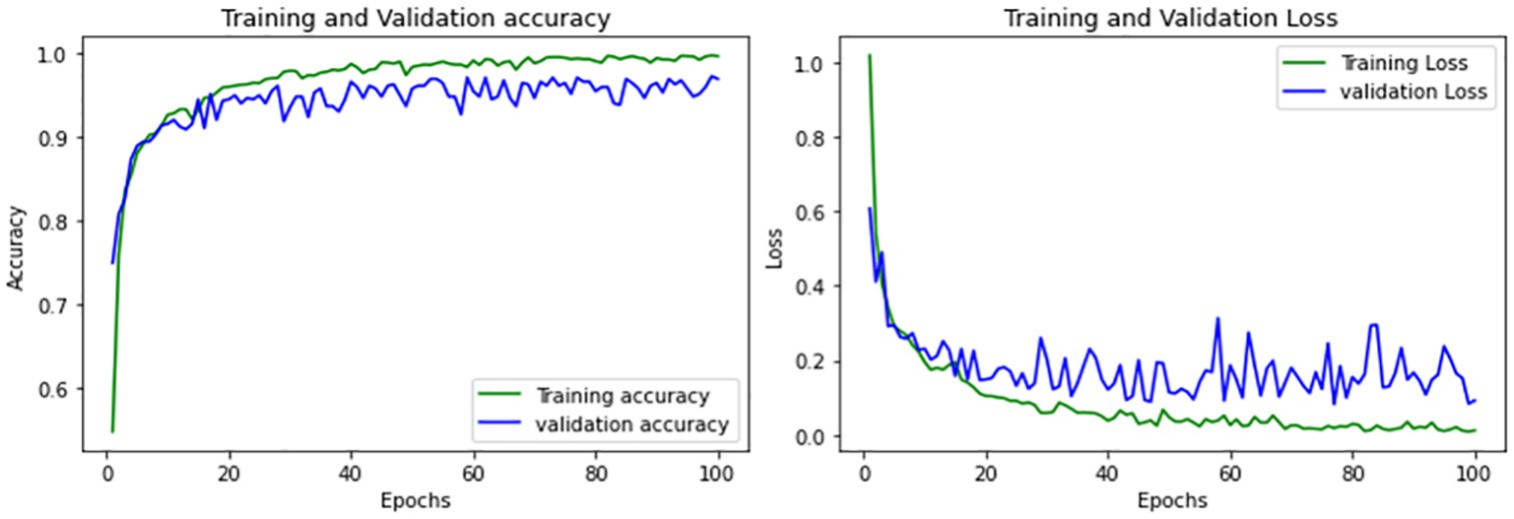
Performance curves of base DenseNet model.

In our brain tumor classification task, the base DenseNet model exhibits excellent performance in comparison to other employed models, by achieving accuracy of 0.96. However, it too faces challenges such as potential overfitting. But DenseNet’s overfitting as can be seen in Fig 8 seems less severe compared to some other implemented models, indicating better generalization capabilities. Due to the commendable performance in comparison to other models, DenseNet is chosen for further experimentation and improvement has been done by incorporating regularization techniques and hyperparameter tuning of the base DenseNet model.

The performance of the DenseNet—Base model is shown in the classification report given in Table 4.

**Table 4:** Classification report of base DenseNet.

Tumor Class	Precision	Recall	F1-score	support
Meningioma	0.92	0.92	0.92	142
Glioma	0.99	0.96	0.98	306
Pituitary	0.93	0.99	0.96	165
Accuracy			0.96	613
Macro Average	0.95	0.95	0.95	613
Weighted Average	0.96	0.96	0.96	613

### Enhanced DenseNet

**Fig 9:**
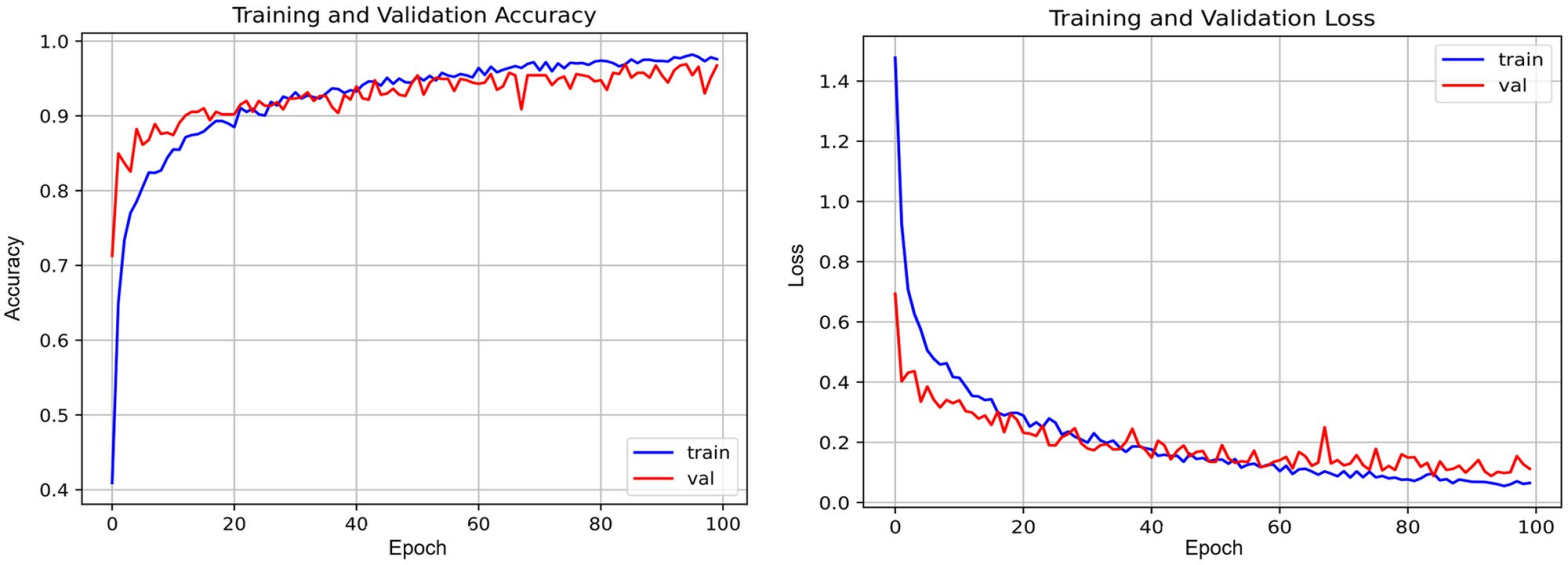
Performance curves of Enhanced DenseNet model.

Our study addressed the overfitting issue observed in the base DenseNet model by employing fine-tuning and various regularization techniques previously discussed. This approach significantly improved the model’s performance on the validation set, as shown in Fig 9. Notably, the learning curves for the Enhanced DenseNet model exhibit minimal divergence up to 100 epochs, indicating excellent generalization to unseen data. In contrast, the base DenseNet model displayed a significant gap between these curves after only 20 epochs, suggesting overfitting.

Table 5 presents the classification report detailing the performance of the Enhanced DenseNet model on the test data.

**Table 5:** Classification report of Enhanced DenseNet.

Tumor Class	Precision	Recall	F1-score	support
Meningioma	0.97	0.91	0.94	142
Glioma	0.98	0.99	0.98	306
Pituitary	0.95	0.99	0.97	165
Accuracy			0.97	613
Macro Average	0.97	0.96	0.97	613
Weighted Average	0.97	0.97	0.97	613

### Performance comparison

**Fig 10:**
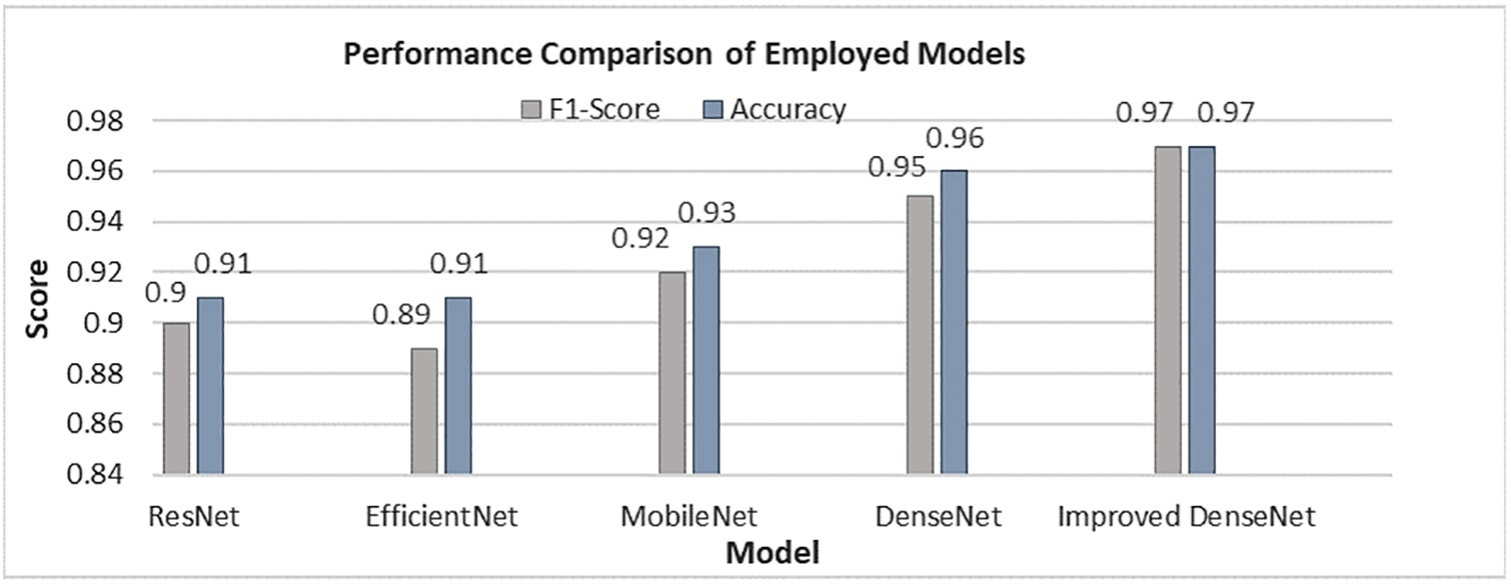
Performance comparison of employed pretrained models.

**Fig 11:**
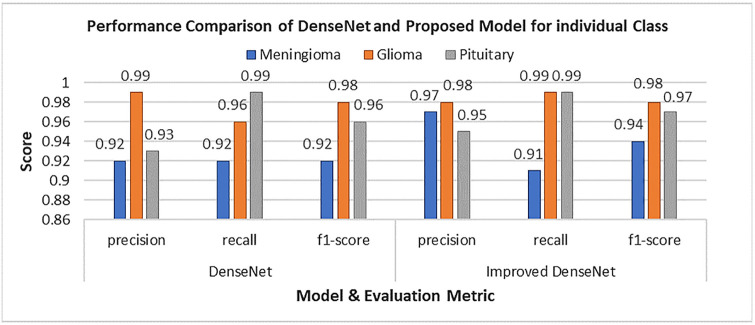
Class-wise performance of base DenseNet and Enhanced DenseNet models.

The accuracy and F1 score performance of all the employed models in this study can be viewed in Fig 10. Additionally, Fig 11 displays the performance of the Base DenseNet and Enhanced DenseNet, for each class in terms of precision, recall and F1 score.

In the case of Meningioma, the model achieved a precision of 0.97, recall of 0.91 and an F1 score of 0.94. This suggests that the model excels in identifying cases while minimizing false positives. Regarding Glioma the model attained a precision of 0.98, a recall of 0.99, and an F1 score of 0.98. showcasing its accuracy and sensitivity in detecting glioma cases with false negatives. For Pituitary tumor classification, the model reached a precision score of 0.95, a recall score of 0.99, and an F1 score of 0.97, indicating its proficiency in categorizing pituitary tumor instances with both high precision and recall rates. The overall model accuracy was documented as 0.971 denoting its classification ability for brain tumor images within the Figshare dataset, with accuracy.

### Comparison with benchmark and similar studies

**Fig 12:**
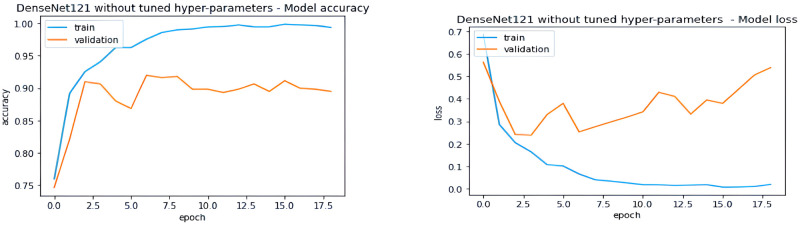
Performance curves of DenseNet model without fine-tuning from benchmark [7].

**Fig 13:**
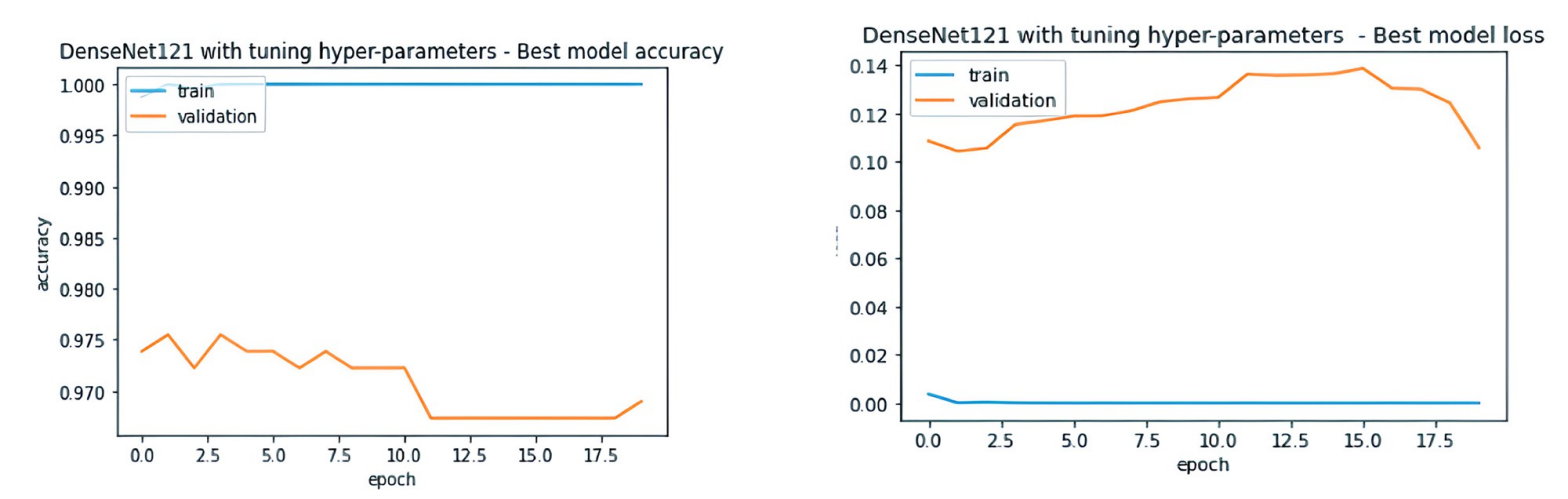
Performance curves of DenseNet model with fine-tuning from benchmark [7].

We compared the performance of our Enhanced DenseNet model with the DenseNet models from the benchmark study by Prakash et al. as illustrated in Figs 12 and 13. The base model graph Fig 12 shows smooth learning over the training set; however, its performance on the validation set is degraded, indicating potential overfitting and an inability to generalize well to unseen data.

In contrast, their fine-tuned DenseNet Fig 13 shows the training accuracy plateauing at 100% from the beginning, while the validation accuracy diverges significantly. This suggests minimal learning and poor generalization capabilities.

These observations clearly demonstrate the substantial performance advantage of our Enhanced DenseNet approach compared to the models presented in the benchmark study.

The comparison of our proposed method with the benchmark and other similar studies in terms of accuracy is also provided in Table 6.

**Table 6:** Comparison with other similar studies.

Study	Technique	Accuracy	Dataset
Abiwinanda et al.(2019) [23]	CNN	84.19	Figshare
Suganthe et al.(2020) [34]	RNN	90	600 MRI Brain Images
Badža et al.(2020) [35]	New CNN	96.56	Figshare
Srinivas et al.(2022) [36]	VGG-16	96	Figshare
Raza et al.(2024) [8]	DenseNet-121	93.70	Public Repository TCGA
Podder et al.(2021) [37]	VGG16, VGG19	86.62, 85.32	253 MR Images
Ghassemi et al.(2020) [4]	GAN+ConvNet	95.6	Figshare
Kaur et al.(2020) [38]	Alexnet	95.92	Figshare
Afshar et al.(2019) [39]	Capsule Networks	90.89	Figshare
Shaik et al.(2022) [40]	Multi-level attention network	96.51	Figshare
Khan et al.(2021) [41]	VGG-16, ResNet50	96, 89	253 brain MRI images
Benchmark (2023) [7]	Enhanced DenseNet	96.5	Figshare
Proposed Method	Enhanced DenseNet	97.10	Figshare

## Conclusion and future research directions

This study investigated the development of a robust and generalizable deep learning model for brain tumor classification using MRI scans. We addressed the limitations of existing approaches, which often prioritize high accuracy on the training data without sufficient emphasis on generalizability to unseen data. We systematically compared DenseNet with other state-of-the-art pre-trained models, including ResNet, EfficientNet, and MobileNet, finding DenseNet to be the most effective on the Figshare dataset. Our enhanced DenseNet model, fine-tuned with data augmentation, dropout, batch normalization, and global average pooling, achieved an impressive overall accuracy of 97.1%. Precision, recall, and F1-scores exceeded 0.94 for all three tumor types: meningioma, glioma, and pituitary tumor, demonstrating the model’s robustness.

Our approach significantly outperformed Base DenseNet and the DenseNet models, presented in the benchmark study, which exhibited substantial overfitting and poor generalization. By incorporating comprehensive model comparison, rigorous fine-tuning, and effective regularization techniques, we developed a highly accurate and generalizable model suitable for clinical settings. This advancement holds promise for enhancing patient care by assisting healthcare professionals in making informed diagnostic decisions for brain tumor detection.

Despite the promising results, our study has several limitations that point towards fruitful avenues for future research. The model’s generalizability to diverse clinical datasets with different MRI protocols needs further validation. Future research should focus on testing larger, varied datasets. Additionally, while using several regularization techniques collectively, their individual impacts were not isolated. Future studies should analyze each technique independently.

We also recommend systematically investigating hyperparameter impacts with techniques like grid search or random search. Addressing these limitations will refine deep learning models for brain tumor classification, enhancing diagnostic accuracy and reliability.
